# Modular continuous wavelet processing of biosignals: extracting heart rate and oxygen saturation from a video signal

**DOI:** 10.1049/htl.2015.0052

**Published:** 2016-04-28

**Authors:** Paul S. Addison

**Affiliations:** Medtronic Respiratory & Monitoring Solutions, Technopole Centre, Edinburgh EH26 0PJ, UK

**Keywords:** wavelet transforms, video signal processing, medical signal processing, feature extraction, cardiology, photoplethysmography, haemodynamics, noisy nonstationary biosignals, acute desaturation, porcine model, video photoplethysmogram, SvidO2, absolute saturation signal, wavelet transform, video signal, oxygen saturation, heart rate extraction, modular continuous wavelet processing

## Abstract

A novel method of extracting heart rate and oxygen saturation from a video-based biosignal is described. The method comprises a novel modular continuous wavelet transform approach which includes: performing the transform, undertaking running wavelet archetyping to enhance the pulse information, extraction of the pulse ridge time–frequency information [and thus a heart rate (HR_vid_) signal], creation of a wavelet ratio surface, projection of the pulse ridge onto the ratio surface to determine the ratio of ratios from which a saturation trending signal is derived, and calibrating this signal to provide an absolute saturation signal (S_vid_O_2_). The method is illustrated through its application to a video photoplethysmogram acquired during a porcine model of acute desaturation. The modular continuous wavelet transform-based approach is advocated by the author as a powerful methodology to deal with noisy, non-stationary biosignals in general.

## 1 Introduction

Remote acquisition of physiological signals from video image streams is attracting much attention in the bioengineering space. In particular, the video photoplethysmogram may be used to derive the two main vital signs displayed on a pulse oximeter device: heart rate (HR) and oxygen saturation (SpO_2_). HR has been tackled by many groups using a variety of signal processing approaches [1–8]. The more challenging task is the determination of oxygen saturation. Kong *et al.* [8] demonstrated a two-camera system for the determination of oxygen saturation based on visible light where two narrow-band filters (at 660 and 520 nm) were mounted on the cameras. They found good agreement with a traditional finger sensor. More recently, Tarassenko *et al.* [9] employed autoregressive modelling and pole cancellation of the red, green, blue (RGB) signals from a digital video camera to determine HR and respiratory rate from patients undergoing a dialysis session. They also demonstrated how desaturation events may be tracked in obstructive sleep apnea patients. The same group have also shown that it is possible to monitor HR, respiratory rate and changes in oxygen saturation in the neonatal intensive care unit [10]. The determination of oxygen saturation using standard video equipment would negate the requirement for specialist equipment and enable the rapid dissemination of such technologies. In the work reported here, a method for extracting HR and saturation trending from a standard video signal (HR_vid_ and S_vid_O_2_) is described which comprises a number of distinct algorithm modules based on the continuous wavelet transform (CWT) [11, 12].

## 2 Method

**Fig. 1:**
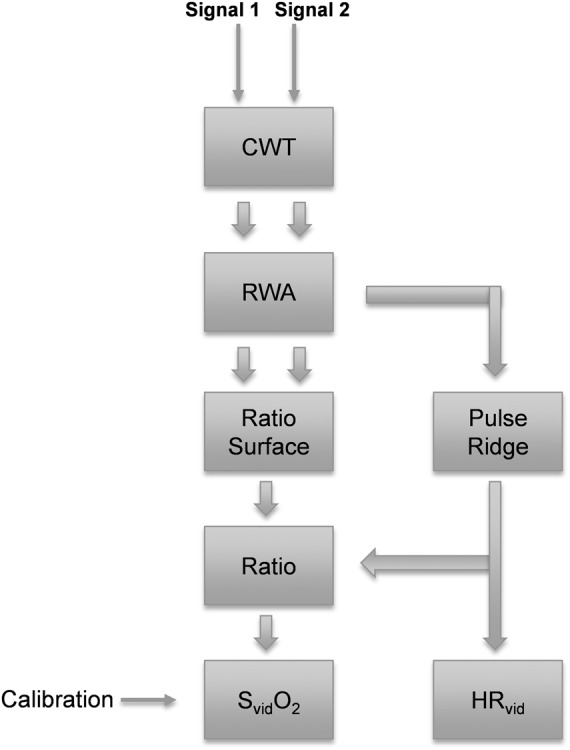
Flow diagram of method for determining oxygen saturation and HR from video signals Two CWTs are computed, one for each input signal, and two RWAs are computed. These are then combined to form a single ratio surface

The flow diagram in Fig. 1 describes the method. Two input signals are used: the *R* and *G* video signals (from RGB). This was found to be the best performing of the three possible two-signal combinations. (Note that others have used the *R* and *B* channels [9, 10]). A CWT, *T*(*a*, *b*), of each signal is first computed

(1) $$T\lpar a\comma \; b\rpar = \displaystyle{1 \over {\sqrt a }}\int_{ - \infty }^{ + \infty } {x\lpar t\rpar \psi ^\ast \left({\displaystyle{{t - b} \over a}} \right){\rm d}t} \eqno\lpar 1\rpar $$

where ψ*(*t*) is the complex conjugate of the wavelet function *ψ*(*t*), *a* is the dilation or scale parameter of the wavelet, *b* is the location parameter of the wavelet and *x*(*t*) is the signal under investigation [11]. A tunable Morlet-based continuous wavelet was employed as it provides the ability to alter the shape of the time–frequency footprints (Heisenberg boxes) of the wavelet in the transform domain [13]. Running wavelet archetypes (RWAs) are then generated from the two transforms. The RWA enables a time–frequency ensemble averaging which takes place in the transform domain and has the advantage over temporal methods of not requiring a knowledge of signal fiducial points [14]. The archetype transform, *T*_RWA_(*a,b*), is generated using a weighted averaging scheme as follows

(2) $$T_{{\rm RWA}}\left({a\comma \; b} \right)= w T\left({a\comma \; b} \right)+ \left({1 - w} \right)T_{{\rm RWA}}\left({a\comma \; b - P\left(a \right)} \right)\eqno\lpar 2\rpar $$

where *w* is a predefined weight, *T*_RWA_(*a*, *b* − *P*(*a*)) is the previous archetype value separated from the current value by a period *P*(*a*) and *T*(*a*, *b*) is the currently computed wavelet transform. In the method, each time a value of *T*(*a*, *b*) is computed, it is used with the previous archetype transform value to form a new value of the archetype transform. As the wavelet transform already separates out the signal information into natural temporal scales according to the periodicity of the wavelet function, the characteristic period of the wavelet may be used at each scale for *P*(*a*)*.* Thus, it can be seen that there is no requirement for the determination of fiducial points in the method, as the wavelet information is naturally ‘rolled up’ at each scale using *P*(*a*) based on the wavelet function itself. The RWA method, fully described in [14], produces a more coherent manifestation of the pulse ridge in the transform domain.

Once the RWA is computed from the CWT, a ridge extraction algorithm is employed to extract the instantaneous frequency of the pulse ridge from the RWA transform. The ridge algorithm detects local maxima of the pulse band with respect to wavelet characteristic frequency. The ridge locus provides the instantaneous video-based HR signal, HR_vid_. In addition, a wavelet ratio surface is computed from the RWAs by dividing the modulus of the two RWA transforms. (This is similar to the method described in [15], but using the RWA transform moduli instead of the original CWT moduli). The ratio surface provides a time–frequency decomposition of all ratios which may be used to compute a value of oxygen saturation. The HR_vid_ ridge locus is projected onto the ratio surface to determine the optimal ratio through time. This ratio is then used to determine the oxygen saturation through calibration with a reference pulse oximeter device (Nellcor N600x, Medtronic, Boulder, CO).

## 3 Monitoring during an acute hypoxic event

**Fig. 2:**
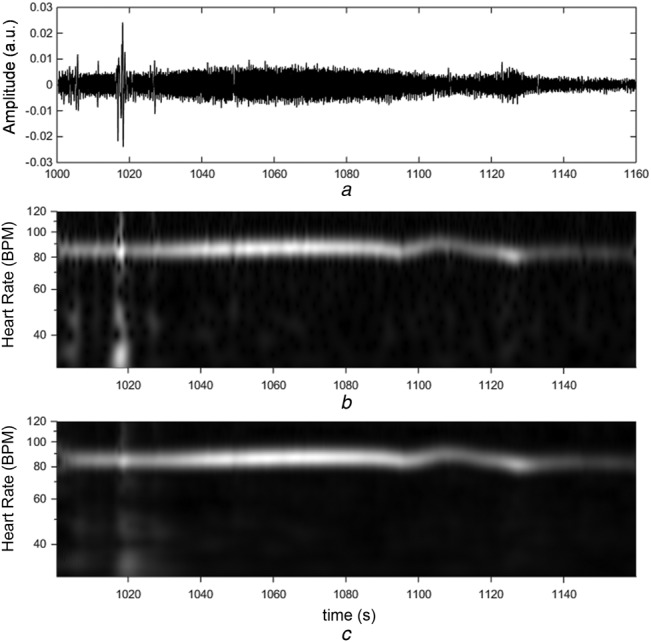
R signal with corresponding scalogram and associated RWA *a R* signal *b* Transform modulus plot *c* Corresponding RWA scalogram

The method described above was applied to a standard video sequence from a commercially available standard RGB video camera (Panasonic HDC-TM10 High Definition Video Camera). Video footage was acquired during a porcine model undergoing a rapid desaturation event where the arterial oxygen saturation dropped from 100% to around 40% for 2 min before being brought back to 100%. A Nellcor pulse oximeter (Medtronic, Boulder, CO) was also attached and provided a reference HR and oxygen saturation. The *R* and *G* signals were extracted from the video image, which was zoomed in on visible skin around the face region. The AC components from each signal were extracted by high-pass filtering at 0.25 Hz to remove respiratory and other lower frequency signal components, and then normalised by their DC component. The normalised AC signals were then wavelet transformed using a Morlet wavelet with characteristic frequency *ω*_0_ = 15 [13]. The *R* signal is shown in Fig. 2*a* with the corresponding wavelet modulus plot in Fig. 2*b*. A distinct pulse band can be seen across the transform modulus plot at around 85 beats per minute. The corresponding processed RWA scalogram is shown in Fig. 2*c*, where the smoothing of the band relative to the original scalogram in Fig. 2*b* is evident.

**Fig. 3:**
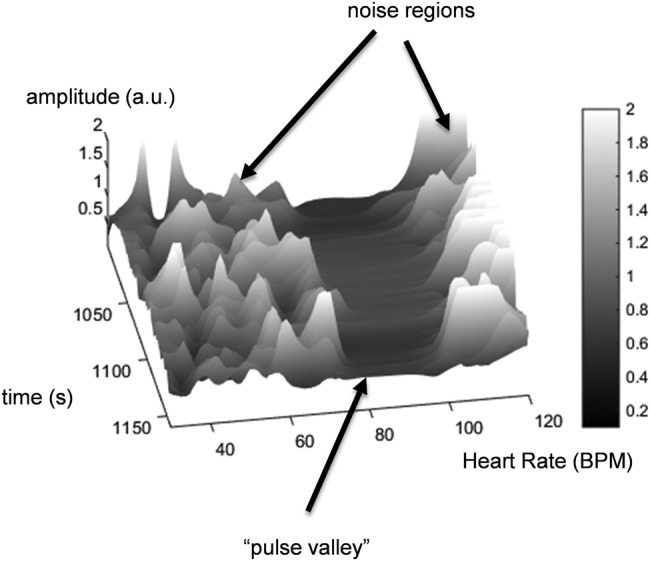
Wavelet ratio surface constructed from two RWAs

**Fig. 4:**
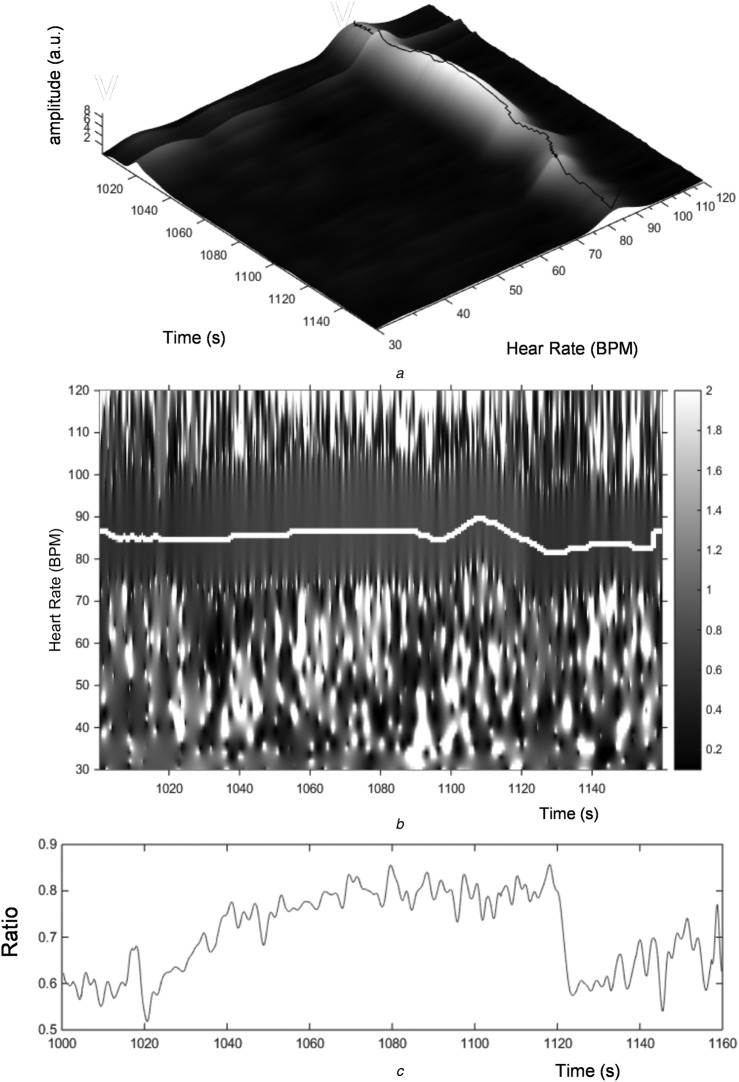
Ratio through time from ratio surface *a* Ridge found from the RWA pulse band maxima *b* Pulse band projected onto the ratio surface shown in Fig. 3 *c* Extracted surface heights (=ratio)

**Fig. 5:**
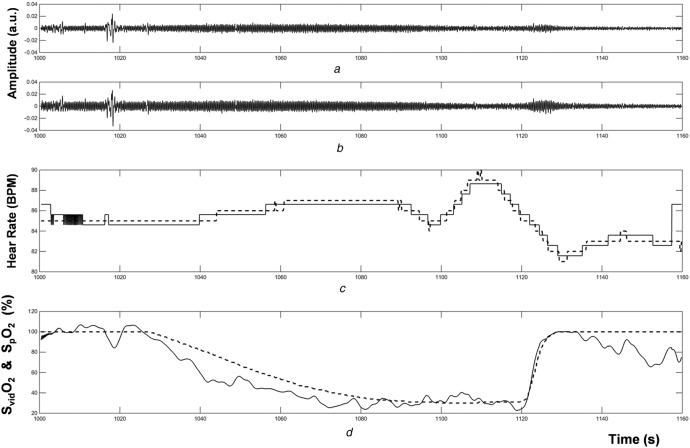
Oxygen saturation *a* Video *R* signal *b* Video *G* signal *c* Dashed line: pulse oximeter HR_p_. Full line: video HR_vid_ *d* Dashed line: pulse oximeter SpO_2_. Full line: video S_vid_O_2_

The wavelet ratio surface derived by dividing the *R* RWA transform modulus by the *G* RWA transform modulus is shown in Fig. 3. There is an obvious flat ‘valley’ in the surface coincident where the two dominant pulse bands divide each other out. Noise manifests on the surface as distinct spikes in the off-pulse band regions. An optimal ratio is extracted through time by simply projecting the pulse rate onto the ratio surface. The pulse rate may be derived by extracting the wavelet ridge frequency (i.e. the video-based HR signal) over time. This is shown in Fig. 4*a*. Apart from localised edge effects, the video and pulse oximeter HRs stay within 2 BPS of each other during the period of investigation (see also Fig. 5*c*). The projection of the pulse rate onto the ratio surface is shown in Fig. 4*b* and the subsequent extracted ratio is shown in Fig. 4*c*. This ratio is then calibrated using the existing oximeter reading. The calibrated video saturation signal is presented in Fig. 5*d* along with the original oximeter-based saturation (SpO_2_) signal. The original *R* and *G* signals and the extracted HRs are also shown in Figs. 5*a*–*c*, respectively. Note that the S_vid_O_2_ appears to fall more rapidly than the SpO_2_. This is a manifestation of the difference in filtering characteristics of the two signals: one is wavelet based, applied to a video signal, and the other uses traditional pulse oximeter algorithmic components and is applied to a photoplethysmogram acquired at the finger.

## 4 Conclusion

A method has been detailed for the computation of HR and oxygen saturation from video signals comprising a series of code modules built around manipulations of the CWT. The method allows for the main vital signs currently measured by a pulse oximeter to be measured using a video system. However, the use of such CWT-based modular approach is advocated by the author as a powerful methodology to deal with noisy, non-stationary biosignals in general. In addition, other modules could be added to further enhance the method. These include reassignment or synchrosqueezing to enhance the definition of the pulse band [16–19], cross-wavelet transforms to measure correlation and phase coupling between the signals as a measure of stability and/or quality [20–23] and secondary wavelet feature decoupling to extract additional (e.g. respiratory) information from the signals [24]. In addition, alternative wavelet-based modules for determining the optimal ratio-of-ratios may be employed; such as one based on the three-dimensional Lissajous method [25].

The signals used in this study came from an opportunistic capture of video during a porcine model of desaturation involving an anesthetised animal where ambient light and motion noise was not an issue. However, significant noise on the signal before and after the hypoxic episode was observed when movement of the investigators in the room caused major fluctuations in ambient light levels. This caused poor-quality signal prior to the desaturation phase and also subsequent to resaturation. In addition, the method as currently set up requires the input of calibration information from an existing oximeter. In practice, this system may be used as a trend and/or event monitor. Alternatively, the absolute saturation value may be calculated in a system that employs intermittent calibration during bedside observation by the caregiver using a pulse oximeter device. In the latter mode, the S_vid_O_2_ approximation of SpO_2_ may be calibrated during spot checks and calculated continuously between these manual checks using the method described here.

In the preliminary investigation described in this Letter, close agreement between the video-derived parameters and pulse oximeter references was observed. A fundamental problem in attempting to compare the ability of video-derived HR and SpO_2_ to track oximeter-derived values is that both ‘devices’ may produce a different time sequence of output values due to internal filtering characteristics and/or internal reporting delays. Thus, matching the two signals may be difficult during rapid changes in the parameter (e.g. as observed in Fig. 5*d*). The study described is, in effect, a tougher challenge than that for a ‘standard’ oximeter hypoxia study [26] where the measured SpO_2_ is altered in discrete steps (e.g. from around 100, to 90, to 80, to 70% then back up again), and where at each step or ‘plateau’ the values are measured only after they become stable. These are then compared with either another device or a reference comprising several blood draws for arterial oxygen saturation values. In contrast, in the study described here, the values were compared dynamically, which is much more challenging and does not lend itself to rigorous comparison. However, this is a consequence of the nature of the study where the data was collected opportunistically and there was no fine control over the SpO_2_ changes.

Future work will include opportunistically adding video capture to other ongoing in-house human hypoxia studies. However, in addition, more specific trials are planned to consider motion and lighting noise in a rigorously controlled environment with reference to the ISO 8060-2-61:2011 (2011) [26] pulse oximeter standard for the evaluation and qualification of pulse oximeter devices.
